# Long-Duration Muscle Dedifferentiation during Limb Regeneration in Axolotls

**DOI:** 10.1371/journal.pone.0116068

**Published:** 2015-02-11

**Authors:** Cheng-Han Wu, Ting-Yu Huang, Bo-Sung Chen, Ling-Ling Chiou, Hsuan-Shu Lee

**Affiliations:** 1 Institute of Biotechnology, College of Bioresources and Agriculture, National Taiwan University, Taipei, Taiwan; 2 Department of Internal Medicine, National Taiwan University Hospital and National Taiwan University College of Medicine, Taipei, Taiwan; 3 Liver Disease Prevention and Treatment Research Foundation, Taipei, Taiwan; 4 Agricultural Biotechnology Research Center, Academia Sinica, Taipei, Taiwan; 5 Research Center for Developmental Biology and Regenerative Medicine, National Taiwan University, Taipei, Taiwan; University of Sheffield, UNITED KINGDOM

## Abstract

Although still debated, limb regeneration in salamanders is thought to depend on the dedifferentiation of remnant tissue occurring early after amputation and generating the progenitor cells that initiate regeneration. This dedifferentiation has been demonstrated previously by showing the fragmentation of muscle fibers into mononucleated cells and by revealing the contribution of mature muscle fibers to the regenerates by using lineage-tracing studies. Here, we provide additional evidence of dedifferentiation by showing that *Pax7* (paired-box protein-7) transcripts are expressed at the ends of remnant muscle fibers in axolotls by using *in situ* hybridization and by demonstrating the presence of Pax7^+^ muscle-fiber nuclei in the early bud and mid-bud stages by means of immunohistochemical staining. During the course of regeneration, the remnant muscles did not progress; instead, muscle progenitors migrated out from the remnants and proliferated and differentiated in the new tissues at an early stage of differentiation. The regenerating muscles and remnant muscles were largely disconnected, and this left a gap between them until extremely late in the late stage of differentiation, at which point the new and old muscles connected together. Notably, *Pax7* transcripts were detected in the regions of muscles that faced these gaps; thus, *Pax7* expression might indicate dedifferentiation in the remnant-muscle ends and partial differentiation in the regenerating muscles. The roles of this long-duration dedifferentiation in the remnants remain unknown. However, the results presented here could support the hypothesis that long-duration muscle dedifferentiation facilitates the connection and fusion between the new and old muscles that are both in an immature state; this is because immature Pax7^+^ myoblasts readily fuse during developmental myogenesis.

## Introduction

Salamanders, including axolotls and newts, exhibit the unique ability to regenerate limbs lost through amputation [1–4]. This regeneration depends on the formation of a blastema tissue containing progenitor cells that regenerate into a new limb at the amputation site [5,6]. Cells in the blastema have been proposed to originate from the dedifferentiation of mature cells, including muscle cells [6–10]. The first evidence supporting this proposal came from histological examinations of the forming blastema that were performed using light and electron microscopy [11,12], which showed the disappearance of myofibrils and the dissociation of muscle fibers into mononucleated cells at the stump. Other experiments conducted by implanting rhodamine-labeled or retrovirally labeled multinucleated myotubes into regenerating newt limbs showed that the labeled myotubes fragmented into mononucleated cells one week later [9,13]. Fluorescent-dye labeling of endogenous tail muscle fibers adjacent to the amputation site also revealed fragmentation of the fibers into mononucleated cells during regeneration [14,15]. Furthermore, 5-bromo-2'-deoxyuridine (BrdU)-incorporation studies demonstrated that retrovirally labeled myotubes reentered the S-phase after implantation [13]. Collectively, these results suggested that mature muscle fibers at the stump dedifferentiate into proliferating progenitors that contribute to the regeneration. However, this theory is still debated. Isolated newt satellite cells, the resident paired-box protein-7 (Pax7)^+^ muscle progenitors, were observed to develop into myotubes in vitro [16] and regenerate into not only muscle but also cartilage during limb regeneration in vivo [17,18]. Recently, a *Cre-loxP* genetic fate-mapping technique was used to investigate whether the labeled mature muscle fibers can contribute to limb regeneration in newts and axolotls [19]. The results obtained added further complexity to the debate: the study showed that muscle fibers contribute to limb regeneration in newts but not axolotls. In axolotls, muscle regeneration probably depends on resident satellite cells.

Based on assuming that dedifferentiated mature muscle fibers must express the transcripts of certain marker genes of muscle progenitor cells, we performed in situ hybridization (ISH) in order to detect the expression of *Pax7* transcripts in axolotl muscle fibers and thereby verify the occurrence of dedifferentiation. Our results revealed that the remnant muscle ends expressed *Pax7* transcripts not only in the early bud and mid-bud stages; the expression also persisted into later differentiation stages. Here, we present a hypothesis developed for explaining the possible role of this long-duration dedifferentiation.

## Materials and Methods

### Animal experimental procedures

Axolotls were maintained in water in a continuous-flow aquarium system at 18–20°C and in a 12/12 h light/dark cycle. The water flowing into the cages was ultraviolet-treated, biofiltered, and adjusted to pH 7.7–8.0. The conductivity of the water was 500–750 μS/cm. Axolotl larvae were fed brine shrimp daily, and adult and juvenile axolotls were fed fish pellets 3 times a week, with leftovers being removed a few hours after feeding. Animal care and experimental procedures were approved by the Institutional Animal Care and Use Committee of the National Taiwan University College of Medicine. Limb amputation of adult axolotls was performed on the upper arms, after which the humerus bones protruded slightly because of the retraction of the surrounding soft tissues. The protruding humerus bones were trimmed to flatten the amputation plane. All surgical experiments were conducted on axolotls that had been anesthetized using 0.1% Tricaine methanesulfonate (MS-222, Sigma-Aldrich, St. Louis, MO, USA).

### Sequencing of axolotl *Pax7* mRNA

**Fig 1:**
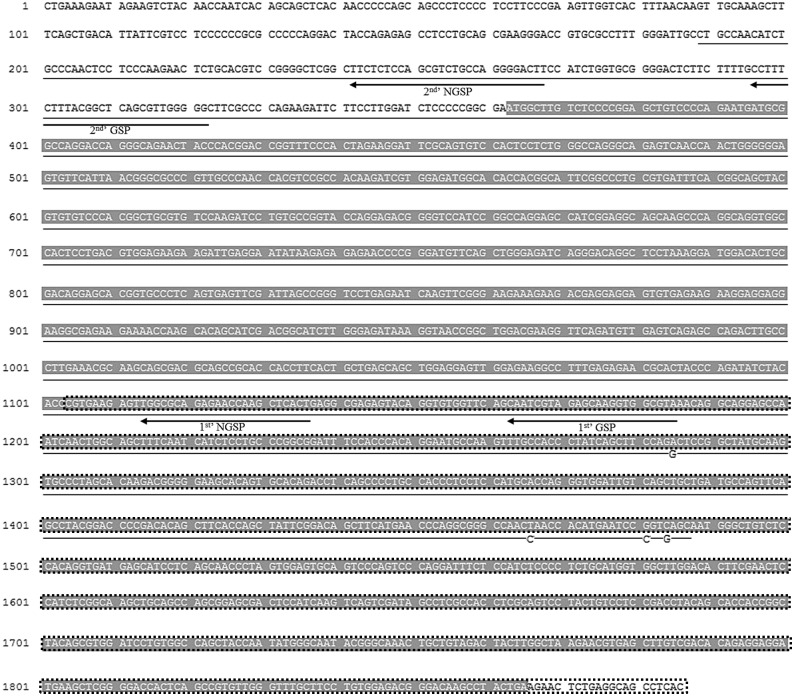
Nucleotide sequence of the axolotl *Pax7* transcript. The underlined sequence has been reported by Schnapp et al. [21]. The 4 underlined nucleotides (nts 1097, 1278, 1293, and 1296) are mismatched with the nucleotides reported by Schnapp et al. The shaded sequence (nt 175–1677) indicates the coding region.

The nucleotide sequence 1104–1886 of axolotl *Pax7* mRNA (Fig. 1) was annotated from our previous next-generation sequencing data [20]. To extend the sequence toward the 5´ end, total RNA was extracted from blastema tissues at the mid-bud stage by using Trizol (Invitrogen, Carlsbad, CA, USA) and 5´-rapid amplification of cDNA ends (RACE) was performed using the SMARTer RACE cDNA Amplification Kit (Clontech, Mountain View, CA, USA). Two rounds of 5´-RACE were performed; the gene-specific primer (GSP) and nested GSP (NGSP) used in each round are indicated in Fig. 1. In the first round, first-strand cDNA was synthesized using SMARTScribe reverse transcriptase and a SMARTer sequence was added at its end, and then RACE PCR was performed using the SMARTer sequence as the forward primer and GSP1 as the reverse primer. The production of an accurate PCR product was further verified by means of nested RACE PCR performed using NGSP1 as the reverse primer, after which the accurate PCR product was subcloned into the pCRII-TOPO vector (Invitrogen) and the insert sequence was determined using an Applied Biosystems automated DNA sequencer (Model 3730, Applied Biosystems, Foster City, CA, USA). Based on the 5´ sequence determined from the first round of RACE, a second round of RACE was likewise performed. The entire obtained sequence was used for identifying homologous sequences in the National Center for Biotechnology Information (NCBI) database by using Basic Local Alignment Search Tool (BLAST). The axolotl Pax7 nucleotide and protein sequences obtained in this study were aligned and compared with previously reported partial axolotl Pax7 sequences [21] and with the sequences of Pax7 in other species by using Vector NTI Suite 12.0 software (InforMax Inc., Frederick, MD, USA).

### Cloning of the coding sequence of axolotl *Pax7* cDNA and preparation of RNA probes

The axolotl *Pax7* mRNA was aligned as described in the preceding subsection; based on this sequence, its coding sequence was cloned using RT-PCR. The total RNA extracted from the mid-bud-stage blastema was reverse transcribed using Superscript II Reverse Transcriptase (Invitrogen). The first-strand cDNA generated was used as the template to amplify *Pax7* mRNA by means of PCR performed using the synthesized forward primer 5´-ATGGCTTGTCTCCCCGGAGC-3´ (nt 363–382; Fig. 1) and reverse primer 5´-TCAGTAGGCTTGTCCCGTCTCC-3´ (nt 1865–1844; Fig. 1). The PCR protocol included denaturing at 94°C for 30 s, annealing at 55°C for 30 s, and extending at 72°C for 1 min. The PCR products were separated electrophoretically in 1.5% agarose gels, and the amplified cDNA in the gels was excised and eluted and cloned into the pCRII-TOPO vector (Invitrogen). From the linearized plasmid, antisense and sense digoxigenin (DIG)-labeled Pax7 RNA probes were transcribed using SP6 and T7 RNA polymerase, respectively, and a DIG RNA-labeling kit (Roche, Indianapolis, IN, USA).

### Whole-mount *in situ* hybridization of *Pax7* transcripts in axolotl embryos

As a positive control for the produced *Pax7* RNA probes, whole-mount ISH in embryos was performed according to the procedure described by Harland [22]. Briefly, axolotl embryos were fixed overnight at room temperature in MEMFA buffer (0.1 M MOPS, pH 7.4, 2 mM EGTA, 1 mM MgSO4, and 3.7% formaldehyde) and treated with proteinase K (20 μg/mL) at 4°C for 30 min and 37°C for 15 min. To reduce the background, embryos were reacted in 0.1 M triethanolamine buffer for 5 min, and to make the tissue transparent, embryos were bleached in 3% hydrogen peroxide. Subsequently, the embryos were hybridized in a prehybridization buffer (50% formamide, 5× SSC, 50 μg/mL yeast RNA, 50 μg/mL heparin, and 1% SDS in 50 mL of DEPC water) containing the probe (5–10 μg/mL) at 60°C overnight. After hybridization, the DIG-labeled probes were detected using anti-DIG-alkaline phosphatase (AP) Fab fragments (Roche), and reaction color was developed using 5-bromo-4-chloro-3-indolyl-phosphate (BCIP) and 4-nitroblue tetrazolium chloride (NBT) dissolved in AP buffer (Sigma-Aldrich). Embryos were cleaned in 70% glycerol for 5 h at room temperature or at 4°C overnight and then photographed.

### Preparation of tissue sections

Regenerating limbs at various stages were cut, embedded in Tissue-Tek (Thermo Scientific, Miami, OK, USA), and frozen at -80°C until sectioning. To obtain longitudinal sections of the remnant muscle tips at all stages, the limb samples were placed horizontally, with the proximal part of the tissue being slightly tilted. However, to obtain the remnant muscle tips and parental muscles continuously in the same sections, the block plane had to be adjusted occasionally by turning the direction of the object-cooling head based on observing the muscle tissues on the sectioning planes during the sectioning procedure. To prepare transverse sections, the regenerating limbs were placed upright and sectioned serially. For use in ISH studies, consecutive 20-μm-thick sections were prepared and used immediately, and for immunohistochemical studies, 7-μm-thick sections were prepared and stored frozen at -80°C until use.

### Tissue-section *in situ* hybridization

Fresh tissue sections were warmed to room temperature and then fixed in cold (4°C) 4% paraformaldehyde in 0.75× PBS for 10 min, after which antisense or sense *Pax7* DIG-labeled RNA probes were hybridized to the sections as previously described [20]. After ISH and incubation with the anti-DIG AP antibody (Roche), color was developed using BCIP/NBT; the samples were briefly washed in 100% ethanol to leach out unbound BCIP/NBT.

### Immunohistochemistry

Cryosections on slides were warmed to room temperature for 10 min and fixed in 1:1 methanol/acetone at -20°C for 5 min. After rinsing in phosphate-buffered saline (PBS) containing 1% Triton X-100 (PBST), the sections were blocked with PBS containing 10% fetal bovine serum at room temperature for 90 min. Subsequently, the sections were incubated overnight (4°C) with primary antibodies against these proteins: Pax7 (1:20 dilution; Developmental Studies Hybridoma Bank, Iowa, USA); and desmin (1:200), myosin heavy chain (MHC; 1:50), and collagen type IV (ColIV, 1:400) (Santa Cruz Biotechnology, Santa Cruz, CA, USA). The sections were then rinsed in PBST, incubated with either fluorescein- or rhodamine-conjugated secondary antibodies (1:200; Jackson ImmunoResearch, Baltimore, MD, USA), counterstained with Hoechst 34580 (Molecular Probes, Eugene, OR, USA), and mounted with fluorescence mounting medium (DakoCytomation, Carpinteria, CA, USA) before being examined using a fluorescence microscope. To detect proliferating cells in tissues, 100 mg/g of 5-ethynyl-2´-deoxyuridine (EdU) was intraperitoneally injected into animals 24 h before harvesting tissues. On the sections, EdU was detected by using the reagents provided in the Click-iT EdU Alexa Fluor 594 Imaging Kit (Invitrogen) according to manufacturer’s instructions. EdU labeling was performed before immunofluorescence staining.

### Microscopy and image processing

Bright-field and fluorescence images were obtained using an Olympus BX51 microscope (Olympus, Tokyo, Japan). The images were processed using Photoshop (Adobe Systems, San Jose, CA, USA) and linear adjustments were performed.

## Results

### Nucleotide and deduced amino acid sequence of axolotl Pax7

**Fig 2:**
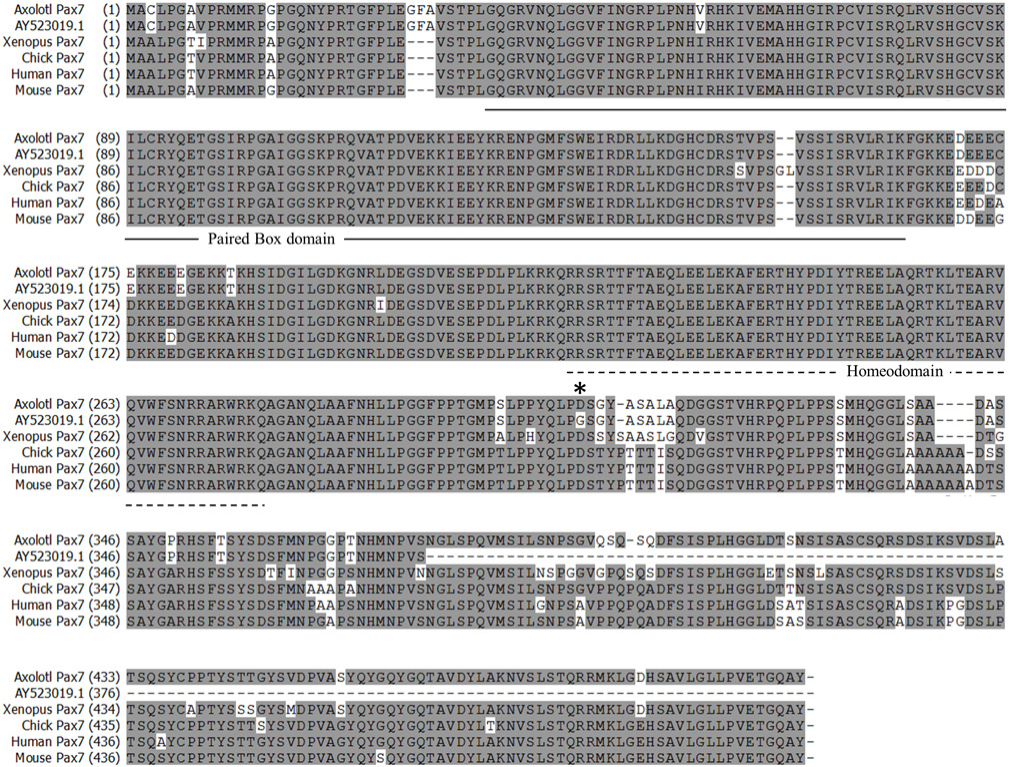
Comparison of the amino acid sequences of axolotl Pax7 deduced from our nucleotide sequence (axolotl Pax7) and from that reported by Schnapp et al. (AY523019.1) with the sequences of Pax7 of other species. The asterisk (aa 308) indicates the only amino acid difference between the sequence reported here and AY523019.1. Axolotl Pax7 and Pax7 from other species exhibit extremely high sequence homology.

In this study, we obtained a 1886-nt sequence of axolotl *Pax7* mRNA (Fig. 1). The cDNA contains a 500-aa open reading frame (nt 363–1865; Fig. 1) that is flanked by a 5´-UTR (362 nts) and a 3´-UTR (21 nts). Compared with the previously published partial cDNA sequence of axolotl Pax7, AY523019.1 [21], the cDNA sequence presented here includes a more complete coding region. Although a 4-nt discrepancy exits between these 2 sequences (nts 1285, 1466, 1481, and 1484), only the nt-1285 discrepancy results in a codon change from aspartic acid to glycine at residue 308, which is also an aspartic acid in the Pax7 sequence in Xenopus, chick, human, and mouse (Fig. 2). The Pax7 amino acid sequences of axolotl, Xenopus, chick, human, and mouse show a high degree of homology: the paired-box domains and homeodomains among these vertebrates are highly conserved, and only a single amino acid difference is present at residue 58 (valine in axolotl, isoleucine in other vertebrates) in the paired-box domain; the homeodomain is completely conserved (Fig. 2).

### Expression of *Pax7* mRNA in axolotl embryos

**Fig 3:**
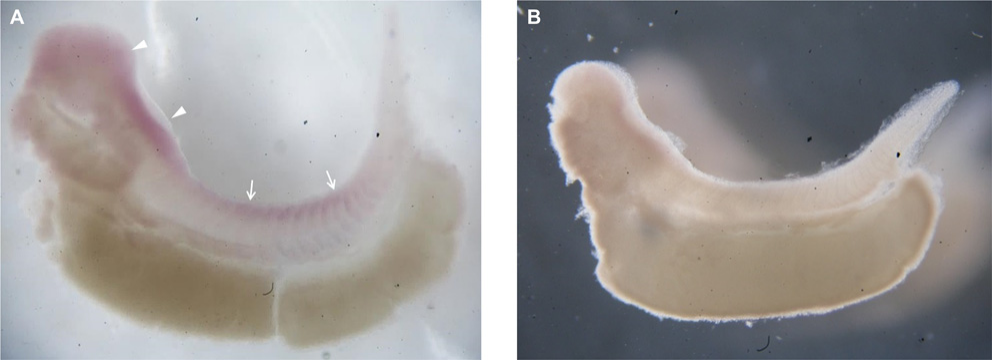
Whole-mount in situ hybridization of *Pax7* in axolotl embryos. (A) An embryo hybridized with the *Pax7* antisense probe shows positive signals in the head, with the signal extending from the forebrain (arrowheads) to the dorsal side of the spinal cord (arrows). (B) A control embryo hybridized with a sense *Pax7* probe shows negative staining.

Whole-mount ISH of *Pax7* in axolotl embryos clearly showed that *Pax7* expression extended from the forebrain and hindbrain to the spinal cord; moreover, we observed a segmented expression pattern indicating somite staining (Fig. 3A), which agrees with the *Pax7* expression pattern in the developing embryos of other vertebrates [23–25]. No signal was generated when hybridization was performed using a sense probe (Fig. 3B). These experiments served as the positive and accuracy controls for the labeled RNA probes.

### Appearance of *Pax7* mRNA expression in the muscle stump during the early bud and mid-bud stages

**Fig 4:**
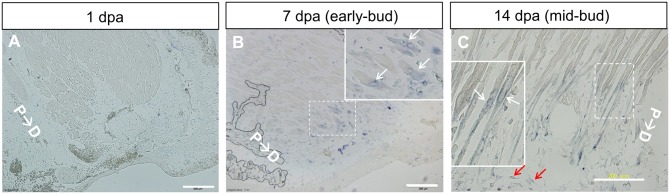
Expression of *Pax7* transcripts in the distal region of remnant muscle fibers at the early bud and mid-bud stages. In situ hybridization of *Pax7* was performed using antisense RNA probes on sections of 1-, 7-, and 14-day-post-amputation (dpa) remnant limbs. (A) No signal was detected on the section of a 1-dpa remnant. (B) On a 7-dpa limb section (early bud stage), sarcoplasm staining can be identified in a few obliquely sectioned muscle cells at the distal end (white arrows). (C) Numerous muscle fibers at the distal remnant of a 14-dpa limb (mid-bud stage) show staining for *Pax7* transcripts in the sarcoplasm (white arrows) and a tapering of the fibers toward the distal parts. Scattered, small *Pax7*-positive cells can be detected in the blastema (red arrows). Insets in B and C are enlarged images of the boxed areas in the same panels. P→D: proximal-to-distal direction. Scale bars = 500 μm in A; 200 μm in B and C.

Longitudinal sections of remnant limbs at 1 day-post-amputation (dpa), 7 dpa (early bud stage), and 14 dpa (mid-bud stage) were examined by performing ISH for *Pax7* expression. No signal was detected in the 1-dpa limb (Fig. 4A). In the early bud stage, a few muscle fibers near the amputation plane were positive for *Pax7* transcripts (Fig. 4B). In the mid-bud regenerates, numerous muscle fibers containing *Pax7* transcripts were detected at the distal ends of the remnant limbs (Fig. 4C). No signal was generated when a sense probe was used for the hybridization (data not shown). At both stages, the *Pax7* transcripts in the muscle stump were present in the sarcoplasm (arrows in Fig. 4B and C). These muscle fibers expressing *Pax7* transcripts became fibrillar and tapered (Fig. 4C). The original muscle fibers proximal to the fibrillar ends were negative for *Pax7* transcripts. Satellite cells could be identified along the length of the muscle fibers. However, the continuity of the *Pax7* ^*+*^ fibrillar ends with the *Pax7* ^-^ proximal fibers strongly argued that these fibrillar ends developed from preexisting muscle fibers instead of from the gathering or linear aligning of activated satellite cells present in this region. The appearance of *Pax7* transcripts in the remnant muscle fibers strongly supported the notion that dedifferentiation had occurred.

### Immunohistochemical staining of muscle ends in longitudinal sections: mid-bud stage

**Fig 5:**
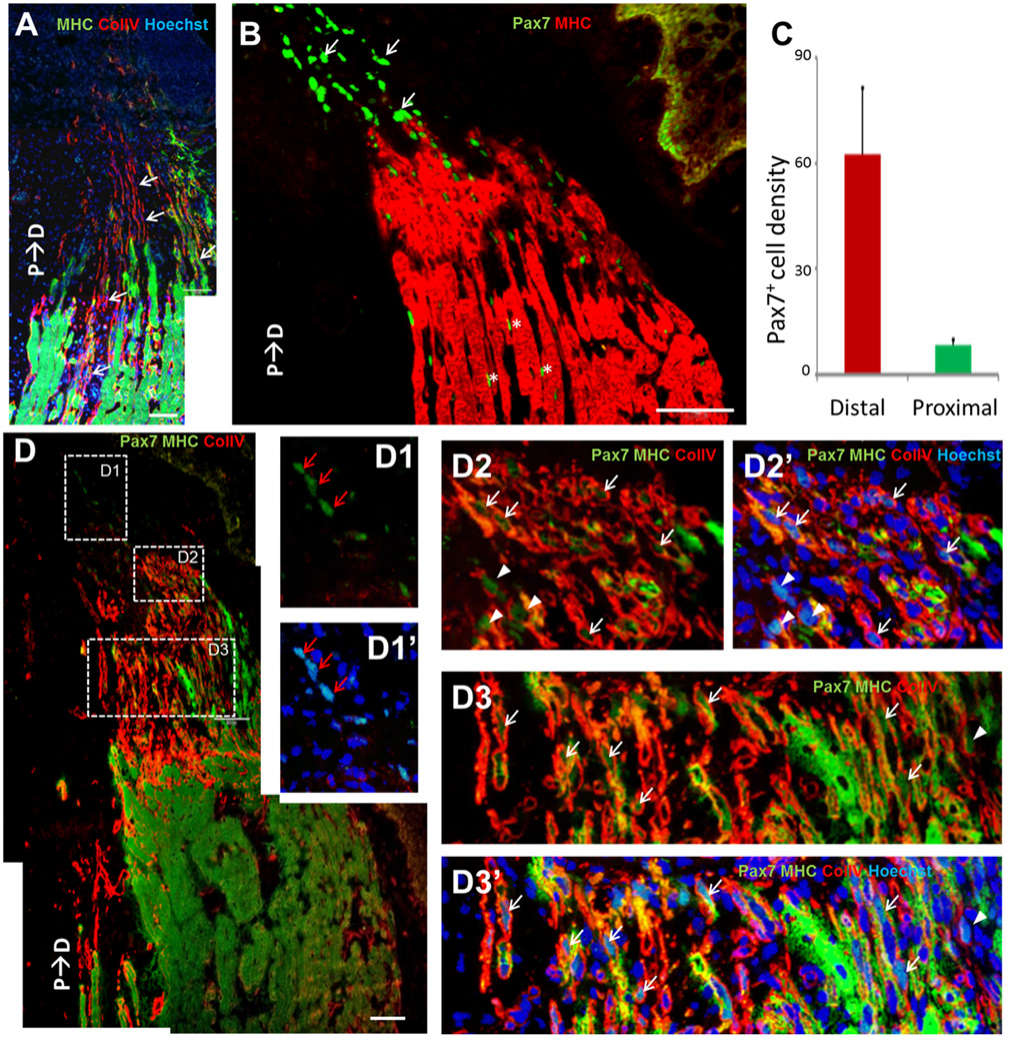
Immunohistochemical staining of remnant muscle ends at the mid-bud stage. (A) Antibody staining of ColIV and MHC was used to demarcate the fibrillar muscle ends that are encircled by a ColIV-containing basal lamina (arrows). The muscle fibers taper and most are MHC^-^. (B) The muscle end shows concentrated Pax7^+^ nuclei (arrows) arranged in a tract away from MHC^+^ muscle fibers and toward the blastema zone. These nuclei are larger and more ovoid than the comparatively more slender nuclei of satellite cells (asterisks) in the proximal muscle. (C) The density of Pax7^+^ nuclei in the distal muscle region is considerably higher than that in the proximal region. (D) Triple staining of Pax7 (green), MHC (green), and ColIV (red) was performed to show Pax7^+^ nuclei present in the fibrillar muscle ends. Nuclei were counterstained with Hoechst in order to differentiate between the Pax7 and MHC signals. D1, D2, and D3 are enlarged images of the respective boxed areas in D. D1´, D2´, and D3´ are images with addition of Hoechst signals in D1, D2, and D3, respectively. Red arrows indicate Pax7^+^ cells distant from the fibrillar ends and extending into the blastema. White arrows indicate Pax7^+^ cells encompassed within a ColIV-containing basal lamina. Arrowheads indicate Pax7^+^ cells partially encircled by ColIV. P→D: proximal-to-distal direction. Scale bars = 100 μm.

The muscle ends in the mid-bud stage were examined by immunolabeling them for ColIV, Pax7, and MHC. ColIV is one of the components of the basal lamina that surrounds muscle fibers. Fig. 5A shows the gradual narrowing of ColIV-encased spaces that were either stained or not stained for MHC (arrows). These narrowed muscle-fiber ends are consistent with the structures of the *Pax7*-expressing fibrillar muscle ends in Fig. 4C. Each ColIV-encased fibrillar end contained linearly arranged cells featuring a scant cytoplasm, and this resulted in a palisade arrangement of nuclei in this region. To determine whether these “palisade-arranged” nuclei belong to muscle progenitor cells, we stained them using an anti-Pax7 antibody. Fig. 5B clearly shows that several palisade-arranged nuclei distal to MHC^+^ muscle fibers were Pax7^+^ (arrows). These nuclei were larger and more ovoid than the typical small and slender satellite-cell nuclei (asterisks) detected in the proximal parts. The density of Pax7^+^ cells was considerably higher in the distal region than in the proximal region (Fig. 5C), which suggested Pax7^+^ cells proliferated actively or that many of the Pax7^+^ cells were recruited from the proximal region. Fig. 5D further shows that the nuclei of several ColIV-encased cells were Pax7^+^. In this region, ColIV encircled these cells either completely (arrows) or only partially (arrowheads) (Fig. 5D2 and D3); by comparison, in the more distal region, linearly arranged Pax7^+^ cells were observed to be completely devoid ColIV encasement (red arrows in Fig. 5D1).

### Immunohistochemical staining of muscle ends in transverse sections: early bud and mid-bud stages

**Fig 6:**
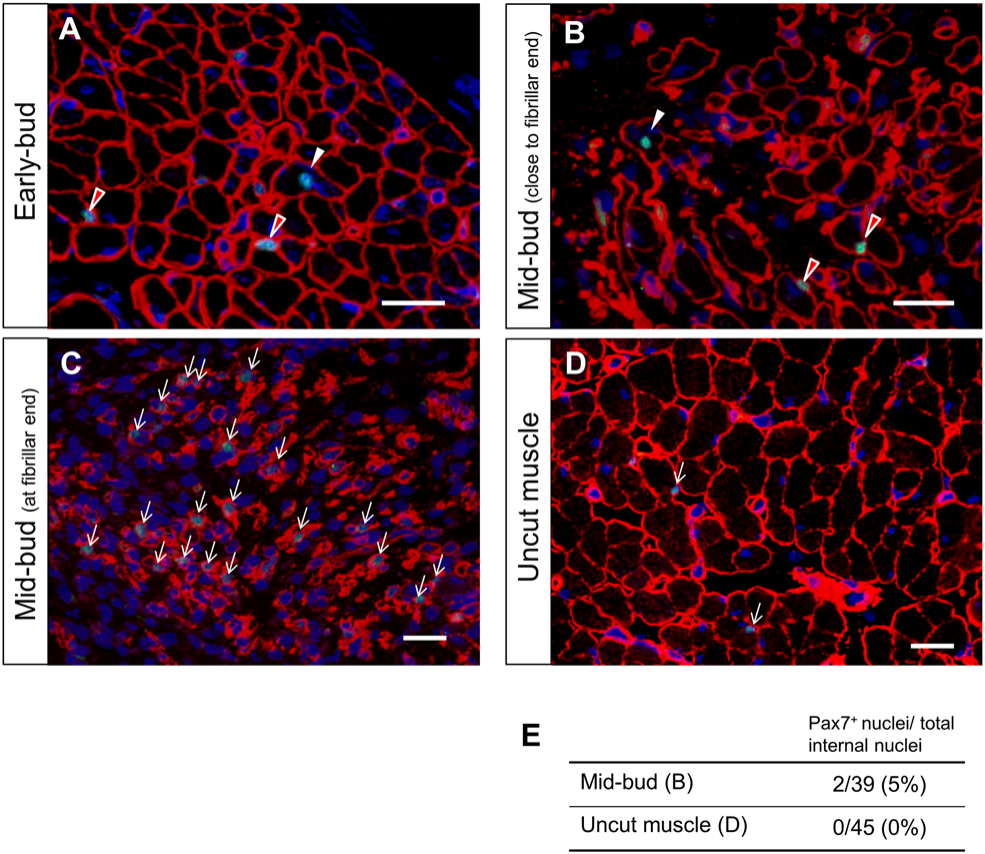
Immunohistochemical staining showing Pax7^+^ muscle-cell nuclei: evidence of dedifferentiation. Transverse sections of the muscle tissue were stained for Pax7 (green) and ColIV (red) and counterstained with Hoechst for nuclei (blue). Each muscle fiber is clearly demarcated by staining for ColIV, which represents basal lamina. At the muscle ends of the remnant limbs at the early bud (A) and mid-bud (B) stages, a few Pax7^+^ nuclei separated from the basal lamina (ColIV) are detected (white arrowheads in A and B). This localization strongly suggests that these are nuclei of muscle fibers. Other Pax7^+^ nuclei lying against the basal lamina (red arrowheads) might represent nuclei of either resident satellite cells or dedifferentiated muscle cells. (C) At the fibrillar ends of a mid-bud remnant, Pax7^+^ nuclei (white arrows) were densely packed within the basal lamina in a manner similar to what was observed in the longitudinal sections. Here, dedifferentiated muscle cells cannot be readily distinguished from the activated resident satellite cells present in this region. (D) A section of an uncut limb at middle arm at a site equal to the amputation plane shows a sparse distribution of Pax7^+^ satellite cells (white arrows). (E) Pax7-positive rates of the internal muscle-fiber nuclei (white arrowhead in B) measured from mid-bud sections (5%; image shown in B) and uncut muscle sections (0%; image in D). Scale bars = 50 μm.

For further verifying the occurrence of muscle dedifferentiation, we performed immunohistochemical staining in order to demonstrate the existence of Pax7^+^ muscle-fiber nuclei. Although preexisting Pax7^+^ satellite cells might also reside in the muscle fibers, these resident satellite cells are located at the periphery of muscle fibers and lie alongside the basal lamina. Our results showed that Pax7^+^ nuclei (white arrowheads in Fig. 6A and B) were present inside the muscle fibers and were separated from the basal lamina (ColIV) in the remnant limbs at the early bud and mid-bud stages. This specific localization pattern strongly suggested that these nuclei belonged to muscle fibers and not to satellite cells. The presence of these Pax7^+^ muscle-cell nuclei indicated the occurrence of dedifferentiation in mature muscle fibers and supported our ISH results. In the fibrillar region of the mid-bud regenerates, Pax7^+^ nuclei were densely distributed in the tapered fibrillar muscle ends (Fig. 6C). In this region, dedifferentiated muscle cells cannot be readily distinguished from activated preexisting satellite cells. In contrast to the observation here, in the muscle of an uncut limb at the middle arm at a site approximately equal to the amputation plane, a typical sparse distribution of Pax7^+^ satellite cells was detected (Fig. 6D). This suggested that the dense distribution of Pax7^+^ cells in the mid-bud remnants was dependent upon amputation. In the sections shown in Fig. 6B, 2 Pax7^+^ nuclei were identified among 39 internal muscle-fiber nuclei. By contrast, among the 45 internal muscle-fiber nuclei detected in the uncut muscle shown in Fig. 6D, none was Pax7^+^ (Fig. 6E).

### Lack of proliferation of Pax7^+^ cells in distal muscle ends at the mid-bud stage

**Fig 7:**
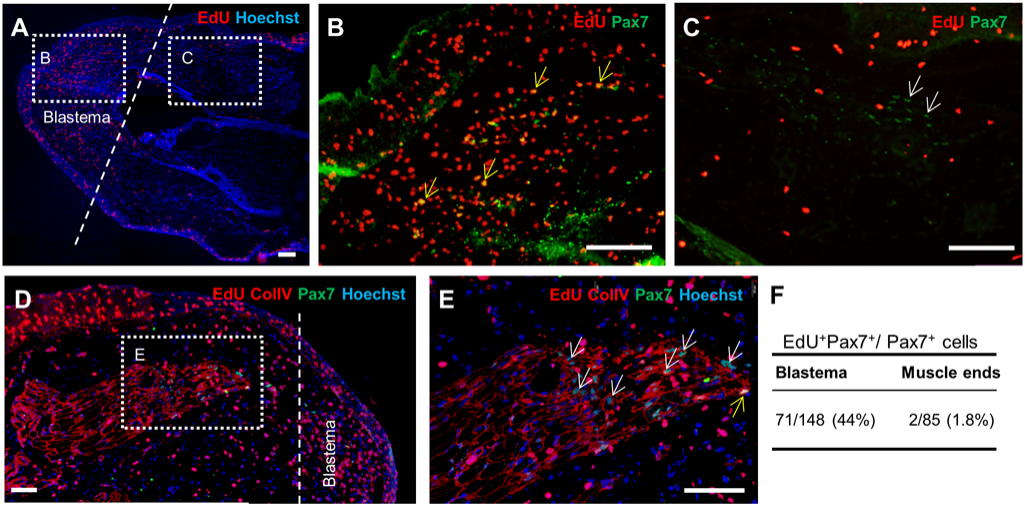
Proliferation of Pax7^+^ cells at the mid-bud stage. Low-magnification views of the sections are shown in A and D. The dashed lines indicate the amputation plane. B, C, and E are enlarged images of the corresponding boxed areas in A and D. (B) Numerous Pax7^+^ cells present in the blastema are EdU^+^ (yellow arrows). (C) Several Pax7^+^ EdU^-^ nuclei exhibit a palisade arrangement (white arrows), which suggests that they are localized in the fibrillar muscle ends. (D) ColIV staining was included to show that the Pax7^+^ cells present within the muscle ends were encircled by a basal lamina. (E) Most of the Pax7^+^ cells in the muscle ends are EdU^-^ (white arrows). The yellow arrow indicates a rare EdU^+^Pax7^+^ cell in the muscle end. (F) A table showing EdU-positive rates among the Pax7^+^ cells present in the blastema and in the muscle ends (n = 4). Scale bars = 200 μm.

To ascertain whether cell proliferation led to the high Pax7^+^ cell density in the distal muscle region, we identified proliferating cells by means of EdU incorporation. Fig. 7B shows that numerous Pax7^+^ cells present in the blastema were EdU^+^ (yellow arrows). However, the palisade-arranged Pax7^+^ cells in the distal regions of remnant muscle were EdU^-^ (white arrows in Fig. 7C). Next, ColIV staining was included to demarcate muscle fibrillar ends (Fig. 7D); in the muscle ends, most of the Pax7^+^ cells were EdU^-^ (Fig. 7E) and extremely few Pax7^+^EdU^+^ cells were observed here. Data pooled from 4 limb samples showed that the EdU-positive rate among Pax7^+^ cells was 44% in blastemas, but only 1.8% in the muscle ends (Fig. 7F). These data indicated that whereas many of the Pax7^+^ cells in the blastema were proliferating, most of these cells in the fibrillar muscle ends were not.

### Muscle regeneration during the early differentiation stage

**Fig 8:**
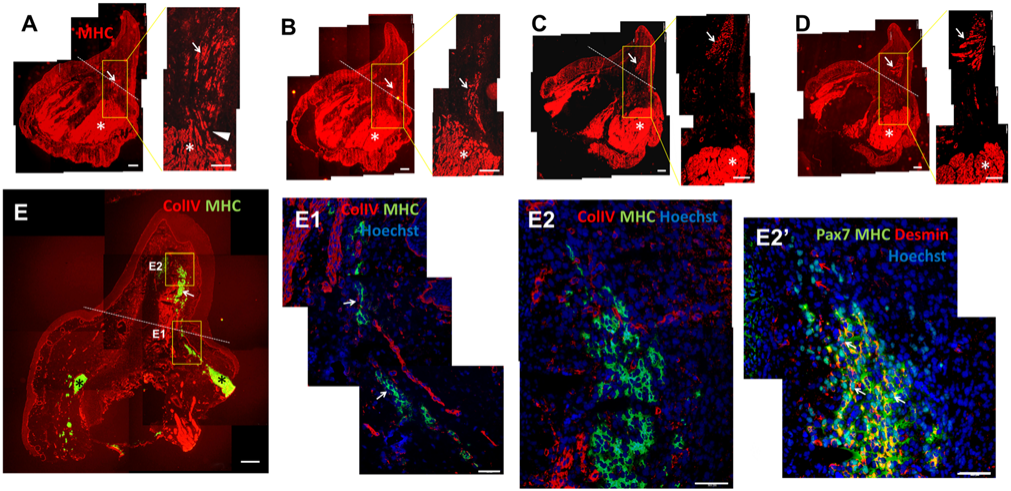
Muscle regeneration during the early differentiation stage. Serial sections (A to D) of a regenerate during the early differentiation stage were stained for MHC. Dashed lines indicate the amputation plane. The regenerating muscles (arrows) are observed to connect only with a projection from the parental muscle (arrowhead in A). (E) A regenerating limb during the early differentiation stage stained for ColIV (red) and MHC (green). The dashed line indicates the amputation plane. (E1, E2) Enlarged images of the boxed area in E. The images show that MHC^+^ muscle cells were not organized into fibers, and that ColIV was not expressed between these immature muscle cells, indicating that the basal lamina had not yet formed by this stage. (E2´) A consecutive section of E stained for Pax7 (green), MHC (green), and desmin (red); the same area as in E2 is shown. MHC and desmin were frequently colocalized. Although several Pax7^+^ cells were MHC^+^ and/or desmin^+^ in the proximal region (white arrows), most of the Pax7^+^ cells in the distal region were negative for MHC/desmin (red arrows), suggesting that muscle differentiation proceeded in a wave from the proximal to the distal region. Asterisks indicate the parental muscle. Scale bars = 200 μm in A, B, C, D, and E; 100 μm in E1, E2, and E2´.

During the early differentiation stage, a few clustered MHC^+^ cells were present in the regenerating limb, and these were mostly separated from the remnant muscle ends and were connected to the muscle ends at a highly limited region; we speculated that the progenitor cells of the regenerating muscle migrated out from the remnants at these connecting regions. Serial sections (Fig. 8A–D) of a regenerating limb in the early differentiation stage clearly showed a limited connecting region between the remnant muscle and the regenerating muscle cells (arrowhead in Fig. 8A). The regenerating MHC^+^ muscle cells tended to be clustered and largely separated from the remnant muscle (Fig. 8B–D). The remnant muscle ends mostly remained a short distance behind the amputation plane, probably because of the physiological retraction of muscle soon after amputation (Fig. 8A–D). During this stage, ColIV was not expressed in the region where MHC^+^ cells were present in the regenerating limbs (Fig. 8E1 and E2), indicating that the basal lamina of the muscle fibers had not yet formed. These new muscle cells were not clearly organized in a fiber-like orientation (Fig. 8E1, E2, and E2´). In the regenerating limb, scattered Pax7^+^ nuclei were frequently observed in the region harboring MHC^+^ cells. Some of the Pax7^+^ nuclei were surrounded by an MHC/desmin-positive cytoplasm (white arrows in Fig. 8E2´), suggesting the presence of differentiating Pax7^+^ muscle progenitors. However, in the leading region of the regeneration, most of the Pax7^+^ nuclei were free of the MHC/desmin cytoplasm (red arrows in Fig. 8E2´). This potentially indicates that satellite-cell differentiation proceeds in a wave from the proximal to the distal part in the regenerating limb.

### Muscle regeneration during the late differentiation stage

**Fig 9:**
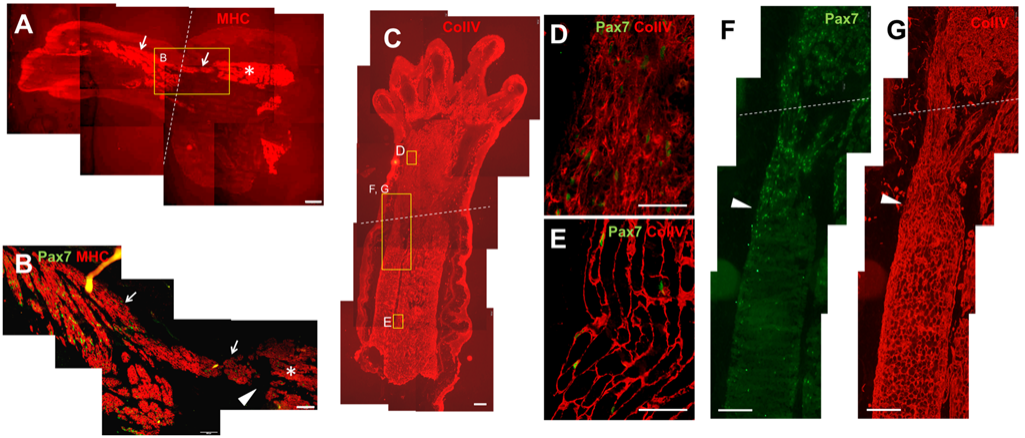
Muscle regeneration during the late differentiation stage. Two limbs are shown in A and C. Dashed lines indicate the amputation plane. White arrows indicate the regenerating muscle, and asterisks indicate the parental muscle. (B) Enlarged image of the boxed area in A; arrowhead indicates a gap between the regenerating and parental muscles. (C) A limb in a late stage of regeneration; the late stage is indicated by the diameter of the regenerating limb being approximately the same as that of the proximal region. D, E, F, and G show enlarged images of the boxed areas in C. The muscle-fiber alignment and ColIV localization here (D) are not as clearly organized as in the proximal region (E); this indicates that the regenerating muscle is still not mature. Arrowheads in F and G indicate the connection between the regenerating and parental muscles. Scale bars = 200 μm in A, B, F, and G; 400 μm in C; 100 μm in D and E.

In the late differentiation stage (Fig. 9A), regenerating muscles were still largely separated from the parental muscle by a gap (arrowhead in Fig. 9B). The remnant muscle ends were again observed to lie slightly behind the amputation plane. The regenerating muscle in this stage was more mature than in the early differentiation stage, which was revealed by the fiber alignment being more organized in this stage than in the early differentiation stage (arrows in Fig. 9B), and also by the expression of ColIV (Fig. 9D). However, the regenerating muscle was not as well organized as mature muscle (Fig. 9E). The limb shown in Fig. 9C is at a considerably late stage of differentiation: the diameters of the regenerated limb and the original proximal region are nearly equal. The regenerated muscle and the parental muscle were extensively connected (arrowheads in Fig. 9F and G), although the regenerated region contained more Pax7^+^ cells.

### Expression of *Pax7* transcripts in the remnant muscle ends during early and late differentiation stages

**Fig 10:**
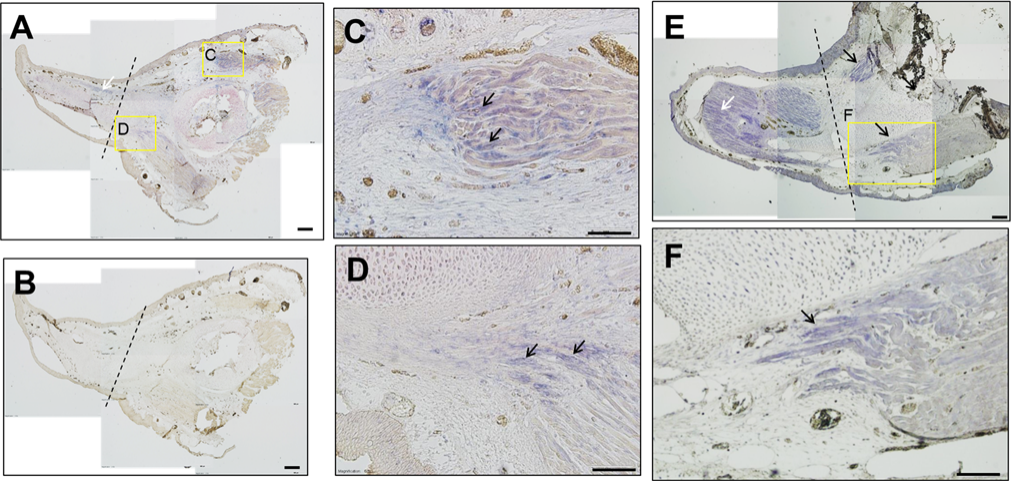
Expression of *Pax7* transcripts in the remnant muscle ends during the early and late differentiation stages. ISH performed using a *Pax7* antisense probe on sections obtained from a regenerating limb during the early (A) and late (E) differentiation stages. (B) Control ISH performed using a sense probe. Dashed lines indicate the amputation plane. (C, D) Enlarged images of the boxed areas in A; positive signals are detected in the remnant muscle ends (black arrows). The white arrow in A indicates a positive signal in the regenerating muscle. No signal is detected in B. (E) A limb during the late differentiation stage. Black arrows indicate positive signals in the remnant muscle ends, and the white arrow indicates the signals in the regenerating muscle. (F) Enlarged image of the boxed area in E; this image clearly shows the positive signal in the remnant muscle fibers. Scale bars = 200 μm in A and B; 100 μm in C, D, E, and F.

Given the observed gradient of *Pax7* transcripts in the remnant muscle ends during the mid-bud stage, we considered it crucial to determine whether the expression persisted until late stages of differentiation. *Pax7* expression persisted until the early (Fig. 10A, C, and D) and late differentiation (Fig. 10E and F) stages and a gradient of *Pax7* expression also present, with more transcripts being detected in the distal regions than in the proximal regions of the remnant muscles (black arrows in Fig. 10C, D, and F). Furthermore, the regenerating muscle fibers also expressed *Pax7* transcripts (white arrows in Fig. 10A and E) during the differentiation stages.

## Discussion

Pax7 is a transcription factor implicated in the specification of myogenic satellite cells [26–28] during the development of skeletal muscle [23,29]. Upon the initiation of myogenic differentiation, the expression of *Pax7* transcripts in satellite cells is rapidly downregulated [30]. Thus, Pax7 is a reliable marker of muscle progenitor cells. We cloned the axolotl *Pax7* cDNA (Fig. 1) and used it as a hybridization probe. As a positive control, whole-mount ISH of axolotl larvae performed using this probe demonstrated *Pax7* mRNA expression in the developing central nervous system (Fig. 3). ISH also showed that in regenerating limbs, *Pax7* mRNA was expressed at the distal ends of the remnant muscle cells at the early bud and mid-bud stages (Fig. 4). During the mid-bud stage, the *Pax7*-expressing muscle fiber ends became fibrillar and progressively thinner and thus indicated a loss of cellular material, as previously demonstrated by means of electron microscopy [2]. Immunohistochemical studies also showed the occurrence of muscle dedifferentiation (Fig. 6) at the early bud and mid-bud stages. In addition to many muscle-fiber nuclei, resident satellite cells are located at the periphery of muscle fibers. Distinguishing satellite-cell nuclei from muscle-cell nuclei in this location can be challenging, although a previous report has shown that newt satellite cells are separated from sarcoplasm by a basal lamina [17]. In this study, we showed that Pax7^+^ nuclei were present selectively at the center of muscle fibers and were separated from the basal lamina (white arrowheads in Fig. 6A and B), and this confirmed their identity as muscle-fiber nuclei. Collectively, our ISH and immunohistochemistry results strongly argue that these muscle ends were dedifferentiating.

Whether or not muscle dedifferentiation occurs during salamander limb regeneration has long been debated. Previous evidence of dedifferentiation was obtained either based on histological observations or by showing the fragmentation of labeled multinucleated myofibers into mononucleated cells [9,31,32]. However, genetic mapping techniques such as *Cre-loxP* systems can be used for convincingly tracing cell fates [33]. Recently, a similar genetic labeling procedure was used on mature muscle cells in order to trace their contribution to regenerated limbs [19]. The results showed that mature muscle fibers contributed to regeneration in newts but not in axolotls, and this potentially suggests that muscle dedifferentiation is not a mainstay in axolotl limb regeneration; however, in the study, the expression of Pax7 or other muscle progenitor-cell markers in the labeled muscle fibers near the amputation plane was not examined [19]. Here, we provide molecular evidence indicating that dedifferentiation occurs in the remnant muscle ends throughout the early bud and mid-bud stages and until the early and late differentiation stages. In a mouse model, *Pax7* was reexpressed in fused myotubes that were tagged using a *Cre-loxP* system, and this was also considered as evidence of muscle cell dedifferentiation after muscle injury [34].

Our data appear to disagree with the results of the genetic tracing experiments that showed that mature muscle cells in axolotls did not contribute to the regenerated limbs [19]. However, our results showed that extremely few Pax7^+^ cells (1.8%) in the fibrillar muscle ends were labeled by EdU during the mid-bud stage (Fig. 7F). This low EdU-labeling rate in Pax7^+^ cells might argue that these dedifferentiated muscle cells did not contribute to the sending out of satellite cells into the regenerating blastema. Furthermore, using immunohistochemistry, we could not readily determine whether the EdU-labeled Pax7^+^ cells in the fibrillar ends (yellow arrow in Fig. 7E) were derived from dedifferentiated cells or from activated preexisting satellite cells. The possibility that these rare cells in the fibrillar ends were derived from activated preexisting satellite cells could not be excluded. Thus, our results are not in conflict with the lineage-tracing studies. By contrast, Kumar et al. [13] determined that in newts, labeled myotubes became BrdU^+^ after implantation and subsequent limb amputation. Thus, our studies in axolotls revealed that dedifferentiated muscle cells might not proliferate, while Kumar’s results in newts showed that dedifferentiated muscle cells could proliferate. This discrepancy between the axolotl and newt studies might agree with the results obtained in lineage-tracing studies [19].

Our results consistently showed that in the early differentiation stage, regenerating muscle tissues were mostly separated from the parental remnant muscle and that only extremely few projections connected them (arrowhead in Fig. 8A). These projections were likely the locations where activated satellite cells had migrated from parental muscles into the regenerating regions. This phenomenon has been observed previously by performing 3D reconstruction of serial sections [35]. In our study, these new regenerating muscle tissues in the early differentiation stage were myoblast clusters that had not yet aligned into myotubes or myofibers (Fig. 8). A wave of muscle-cell differentiation might spread from proximal to distal regions (Fig. 8E2´), and the tract-arrangement of the regenerating muscle tissues (Fig. 8E and E1) indicated that the migration of these new muscle cells must be tightly regulated. The basal lamina had not yet formed at this stage because ColIV immunolabeling was not detected in the muscle-regenerating regions (Fig. 8E1 and E2). However, during this early differentiation stage, *Pax7* mRNA expression was observed at the muscle-remnant ends and in the regenerating muscle (Fig. 10A, C, and D).

New regenerating muscle fibers and old parental mature muscle fibers were observed to be separated by gaps even during the late differentiation stage, by which time the new regenerating muscle fibers had matured considerably (Fig. 9B and D). However, at substantially late stages of the late differentiation, the regenerating and parental muscle fibers were connected to each other (Fig. 9C, F, and G). The induction of ColIV expression in the regenerating muscle regions indicated the development of a basal lamina (Fig. 9G). *Pax7* transcripts were still detected at this stage in both the remnant-muscle ends and the regenerating muscle fibers (Fig. 10E and F). Positive labeling for *Pax7* transcripts might indicate dedifferentiation in the remnant-muscle ends and partial differentiation in the regenerating muscle fibers.

The previous hypothesis on the role of dedifferentiation during salamander limb regeneration was that dedifferentiated muscle progenitors contribute to the regeneration [11,12]. However, previous lineage-tracing studies [19] and our results in Fig. 7 suggest that this scenario might not apply in the case of axolotls. What might be the role of dedifferentiation during limb regeneration in axolotls?

Based on the results showing that the regenerating and parental muscle tissues were separated but connected at a late stage of regeneration, and that dedifferentiation persisted in the remnant-muscle ends and partial differentiation existed in the regenerating muscle fibers, we hypothesized that long-duration muscle dedifferentiation during axolotl limb regeneration occurs mainly for facilitating the connection between new and parental muscle fibers in order to restore the muscle bundles to their preamputation states. During skeletal myogenesis, Pax7^+^ myoblasts are fused into myotubes [36]; therefore, the Pax7^+^ immature status of the muscle-fiber ends facing each other across the gap at late differentiation stages might allow them to fuse together. However, expression of *Pax7* in regenerating muscle fibers is intriguing because according to the paradigm of myogenesis during development, *Pax7* expression is already turned off once myotubes have formed and during subsequent stages [37].

It will be of interest to determine how the parental and the newly formed muscle fibers connect and then fuse together end-to-end in such an accurate alignment. Understanding the underlying molecular and cellular mechanisms of this accurate connection and fusion is biologically interesting; however, this understanding might also help tissue engineers in developing methods to effectively generate a host-implant interface in order to improve tissue integration and enhance healing.
